# Rates and Predictors of Persistent LUTS Medication Use After Laser Enucleation of the Prostate

**DOI:** 10.1111/luts.70062

**Published:** 2026-04-23

**Authors:** Maximilian Filzmayer, Miriam I. Traumann, Clara Humke, Matthias J. Müller, Philipp C. Mandel, Luis A. Kluth, Andreas Becker, Felix K.‐H. Chun, Marina Kosiba

**Affiliations:** ^1^ Department of Urology Goethe University Frankfurt, University Hospital Frankfurt am Main Germany; ^2^ Martini Clinic Prostate Cancer Center University Hospital Hamburg‐Eppendorf Hamburg Germany; ^3^ Department of Urology Asklepios Paulinen Clinic Wiesbaden Germany; ^4^ Urological Center at Boxberg Neunkirchen Germany

**Keywords:** benign prostate syndrome, laser enucleation of the prostate, lower urinary tract symptoms, medication

## Abstract

**Objectives:**

To assess rates of medication use for lower urinary tract symptoms (LUTS) over time following laser enucleation of the prostate (LEP) and to identify preoperative predictors of persistent use.

**Methods:**

We retrospectively analyzed 864 LEP patients from an institutional tertiary‐care database (11/2017–05/2023) with available 24‐month follow‐up on medication use. Patient‐reported use of five drug classes (alpha‐blockers, 5‐alpha‐reductase (AR)‐inhibitors, anticholinergics, beta‐3‐agonists, and phosphodiesterase (PDE)‐5‐inhibitors) was recorded preoperatively and at one, three, 12, and 24 months after the procedure. Univariable and multivariable logistic regression models were fitted to identify predictors of persistent LUTS medication use.

**Results:**

Preoperatively, 84.9% of patients used LUTS medication, decreasing to 12.6% within 24 months of follow‐up. Alphablocker (80.9%) and 5‐AR‐inhibitor (13.8%) use declined to 1.8% and 0%, respectively. Anticholinergic (4.6%) and beta‐3‐agonist (0.3%) use showed a transient postoperative increase with peaks of 10.9% and 1.0% at 3 months, followed by a decline to 2.7% and 0.4% at 24 months, respectively. PDE‐5‐inhibitor use (1.8%) increased steadily to 5.4% at 24 months. Patient with persistent use exhibited worse baseline QoL and ICIQ‐SF scores and higher rates of adiposity, diabetes mellitus, and ASA score III/IV. In multivariable analysis, only preoperative PDE‐5‐inhibitor use (adjusted OR 3.26, *p* = 0.002) and ASA score III/IV (adjusted OR 2.08, *p* = 0.016) remained independently associated with persistent LUTS medication use.

**Conclusion:**

LUTS medication use decreased substantially after LEP, with only a small subset requiring continued medication at 24 months. Preoperative PDE‐5‐inhibitor use and higher comorbidity burden emerged as independent predictors of persistent LUTS medication use. These findings can refine preoperative counseling regarding postoperative LUTS medication dependence.

## 1 Introduction

Benign prostatic hyperplasia (BPH) is a prevalent cause of lower urinary tract symptoms (LUTS) in aging men, and surgical treatment is recommended when medical therapy fails or complications of bladder outlet obstruction occur [1]. Laser enucleation of the prostate (LEP) has proven to be a safe and effective minimally invasive surgical treatment option [1, 2, 3]. It can be performed regardless of prostate size and with less morbidity as compared to transurethral resection of the prostate (TURP) and open simple prostatectomy [2, 3].

For many patients, a central motivation to undergo surgical treatment is the expectation of becoming independent from LUTS medication, namely alphablockers, 5‐alphareductase (AR)‐inhibitors, anticholinergics, beta‐3‐agonists, and phosphodiesterase (PDE)‐5‐inhibitors [4]. However, several studies report notably high rates of persistent LUTS medication use after TURP [5, 6, 7, 8]. For example, Campbell et al. and Ory et al. reported continued prescription of alphablockers in 26% and 15% of patients, respectively [5, 7]. However, the available evidence for LEP is highly heterogeneous, with reported rates of persistent use of alphablockers in the second year after the procedure ranging from as low as 7% in the cohort analyzed by Ory et al. to as high as 34% in the population examined by Sabharwal et al., for instance [6, 7]. To the best of our knowledge, no previous study has reported longitudinal rates of LUTS medication use after LEP, particularly not with separate depiction of the individual pharmacological drug classes.

Regarding predictors of postoperative LUTS medication use after LEP, there is only one Korean study by Kim et al. examining use of anticholinergics and beta‐3‐agonists in 257 patients [9]. These two drug classes are frequently initiated de novo in the early postoperative period to mitigate transient irritative symptoms after the procedure [9, 10, 11]. In addition, Kim et al. did not assess alpha‐blockers, 5‐AR‐inhibitors, and PDE‐5‐inhibitors.

Therefore, there is still limited evidence on LUTS medication use after LEP and its preoperative predictors. To fill this void, we relied on our institutional tertiary‐care database of patients treated with LEP.

## 2 Methods

### 2.1 Patient Cohort

We relied on our prospectively maintained institutional tertiary‐care database to retrospectively identify patients who underwent LEP for LUTS between 11/2017 and 05/2023 and had available data on postoperative LUTS medication use. Patients undergoing palliative LEP for prostate cancer (*n* = 56) were excluded. The final analytical cohort comprised 864 patients. The surgeries were performed by seven surgeons, including three senior LEP experts (caseload over 200), while the others reached proficiency through a structured mentoring program that was previously investigated and maintained high‐quality outcomes throughout the learning curve [12]. The study adhered to the Declaration of Helsinki, with ethical approval from the local Ethical Committee (2021‐171, E 98/21) and written informed consent from all participants.

### 2.2 Outcomes of Interest

Use of LUTS medication was part of patient reported outcome measures collected preoperatively and after one, three, 12 and 24 months [13]. Five pharmacological drug classes were examined: alpha‐blockers, 5‐AR‐inhibitors, anticholinergics, beta‐3‐agonists and PDE‐5‐inhibitors. Only continuous use of long‐acting PDE‐5‐inhibitors was considered, whereas on‐demand intake was not included, as the latter typically reflects treatment of erectile dysfunction rather than management of LUTS. In the current study, the primary outcome was the identification of predictors for persistent LUTS medication use after 24 months of follow‐up. For this analysis, missing longitudinal medication data were handled using a last‐observation‐carried‐forward (LOCF) approach, whereby the last observed medication status for each pharmacological drug class was carried forward to subsequent time points if data were missing [14]. The secondary outcome was the proportion of patients using LUTS medication over time, assessed separately across the five pharmacological drug classes without applying LOCF.

### 2.3 Statistical Analysis

First, rates of use for each of the five drug classes were plotted separately over the follow‐up period to examine the temporal evolution of postoperative medication patterns. Second, preoperative differences between patients with and without persistent use were first assessed using the Wilcoxon rank‐sum test for continuous variables and Pearson's chi‐squared test or Fisher's exact test for categorical variables, as appropriate. Third, univariable logistic regression models (LRM) were fitted for all relevant clinical and functional parameters to evaluate potential predictors of persistent LUTS medication use. Finally, variables demonstrating statistical significance in univariable analysis were subsequently entered into a multivariable LRM to identify independent predictors, following established recommendations for purposeful variable selection [15, 16]. Given the dual indication of PDE‐5‐inhibitors, a sensitivity analysis without accounting for PDE‐5‐inhibitor use was performed [17, 18]. Specifically, postoperative use of PDE‐5‐inhibitors was excluded from the outcome definition, and preoperative use of PDE‐5‐inhibitors was not included as a covariate in the multivariable LRM.

Effect estimates are reported as odds ratios (OR) with corresponding 95% confidence intervals (CI), and statistical significance was defined as *p* < 0.05. All statistical analyses were performed using R (R Foundation for Statistical Computing, Vienna, Austria, RRID: SCR_001905, version 4.4.1). This study was conducted in accordance with the Strengthening the Reporting of Observational Studies in Epidemiology (STROBE) guidelines [19].

## 3 Results

### 3.1 Study Population and Use of LUTS Medication Over Time

**FIGURE 1:**
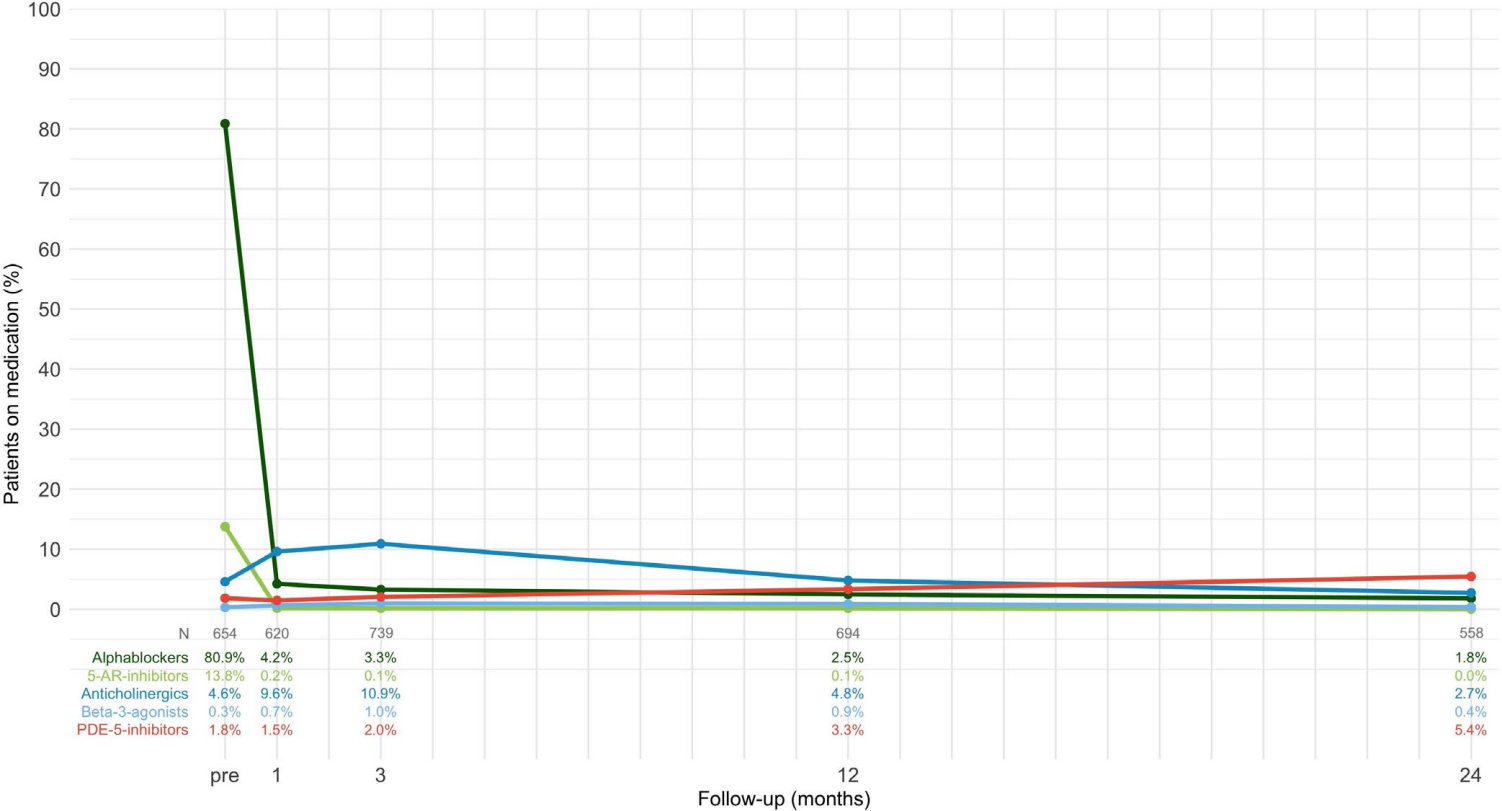
Use of LUTS medication over 24 months long follow‐up in 864 patients undergoing laser enucleation of the prostate at the University Hospital Frankfurt in the period from 11/2017 to 05/2023. AR = alphareductase; PDE = phosphodiesterase; pre = preoperative.

Baseline characteristics of the study population are shown in Table 1. The proportions of patients using the five different pharmacological drug classes of LUTS medication over the follow‐up period of 24 months are shown in Figure 1. Preoperatively, 84.9% of the patients reported use of LUTS medication vs. 12.6% at 24 months after the operation. At baseline, alphablockers (80.9%) and 5‐AR‐inhibitors (13.8%) predominated, while anticholinergics (4.6%), beta‐3‐agonists (0.3%), and PDE‐5‐inhibitors (1.8%) were relatively rare. Postoperatively, the use of alphablockers and 5‐AR‐inhibitors decreased sharply to 4.2% and 0.2% at 1 month, followed by a continued decline at three and 12 months, reaching 1.8% and 0% at 24 months. Use of anticholinergics and beta‐3‐agonists showed a transient postoperative increase, with peaks of 10.9% and 1.0% at 3 months, before declining to 4.8% and 0.9% at 12 months and ultimately to 2.7% and 0.4% at 24 months, respectively. Use of PDE‐5‐inhibitors increased steadily over time, rising from 1.5% at 1 month to 2.0% at 3 months, 3.3% at 12 months, and reaching 5.4% at 24 months.

**TABLE 1:** Preoperative characteristics of 864 patients undergoing laser enucleation of the prostate at the University Hospital Frankfurt in the period from 11/2017 to 05/2023, stratified according to persistent LUTS medication use after 24 months long follow‐up.

Preoperative characteristics	*N*	Overall, *n* = 864	Persistent LUTS medication use, *n* = 109 (12.6%)	No persistent LUTS medication use, *n* = 755 (87.4%)	*p* ^b^
Age (in years), median (IQR)	864	70 (63–75)	70 (62–76)	69 (64–75)	0.836
Prostate volume (in ccm), median (IQR)	854	80 (55–105)	77 (53–114)	80 (56–104)	0.648
PSA serum level (in ng/mL), median (IQR)	749	4 (2–8)	4 (2–7)	4 (3–8)	0.246
IPSS storage, median (IQR)	480	9 (6–12)	10 (7–12)	9 (6–11)	0.070
IPSS voiding, median (IQR)	480	10 (7–14)	11 (8–14)	10 (7–14)	0.243
QoL, median (IQR)	695	4 (3–5)	5 (4–5)	4 (3–5)	**0.001**
ICIQ‐SF, median (IQR)	630	4 (0–9)	6 (0–11)	3 (0–9)	**0.026**
IIEF‐5, median (IQR)	529	11 (4–20)	12 (4–20)	11 (4–20)	0.554
Peak urinary flow rate (in mL/s), median (IQR)	538	10 (7–13)	10 (7–13)	10 (7–13)	0.415
Postvoid residual volume (in mL), median (IQR)	559	100 (45–200)	100 (40–200)	100 (45–200)	0.674
Adiposity, *n* (%)	864	125 (14.5%)	24 (22.0%)	101 (13.4%)	**0.017**
Indwelling catheter, *n* (%)	802	248 (30.9%)	21 (20.4%)	227 (32.5%)	**0.013**
Use of at least one pad per day, *n* (%)	347	293 (84.4%)	41 (87.2%)	252 (84.0%)	0.570
Incontinence,^a^ *n* (%)	501	142 (28.3%)	23 (32.9%)	119 (27.6%)	0.366
ASA risk score III/IV, *n* (%)	864	251 (29.1%)	47 (43.1%)	204 (27.0%)	**< 0.001**
Diabetes mellitus, *n* (%)	628	87 (13.9%)	21 (24.1%)	66 (12.2%)	**0.003**
Use of LUTS medication, *n* (%)	654	555 (84.9%)	80 (90.9%)	475 (83.9%)	0.051
Alphablockers	654	529 (80.9%)	76 (86.4%)	453 (80.0%)	0.160
5‐AR‐inhibitors	654	90 (13.8%)	14 (15.9%)	76 (13.4%)	0.530
Anticholinergics	654	30 (4.6%)	7 (8.0%)	23 (4.1%)	0.106
Beta‐3‐agonists	654	2 (0.3%)	1 (1.1%)	1 (0.2%)	0.251
PDE‐5‐inhibitors	654	12 (1.8%)	4 (4.5%)	8 (1.4%)	**< 0.001**
Surgeon's caseload, median (IQR)	864	228 (98–435)	258 (97–446)	222 (98–432)	0.432
Caseload over 200, *n* (%)	864	474 (54.9%)	67 (61.5%)	407 (53.9%)	0.138

*Note:* Statistically significant values (*p* < 0.05) are shown in bold.

Abbreviations: AR = alphareductase; ASA = American Society of Anesthesiologists; ICIQ‐SF = International Consultation on Incontinence Questionnaire Short Form; IIEF‐5 = International Index of Erectile Function; IPSS = International Prostate Symptom Score; IQR = interquartile range; LRM = logistic regression model; LUTS = lower urinary tract symptoms; PDE = phosphodiesterase; PSA = prostate‐specific antigen; QoL = quality of life.

^a^ Continence defined as ICIQ‐SF ≤ 4 and no more than one security pad per day.

^b^ Wilcoxon rank sum test; Pearson's Chi‐squared test; Fisher's exact test.

### 3.2 Persistent Use of LUTS Medication

Of the 864 patients, 109 (12.6%) exhibited persistent LUTS medication use after 24 months of follow‐up. Patients with persistent LUTS medication use reported significantly worse preoperative QoL (quality of life; median 5 vs. 4) and ICIQ‐SF (International Consultation on Incontinence Questionnaire Short Form; median 6 vs. 3) scores, were less likely to have an indwelling catheter (20.4% vs. 32.5%), showed a higher burden of comorbidities (adiposity: 22.0% vs. 13.4%; ASA [American Society of Anesthesiologists] risk score III/IV: 43.1% vs. 27.0%; diabetes mellitus: 24.1% vs. 12.2%), and more frequently used PDE‐5‐inhibitors (4.5% vs. 1.4%). No preoperative differences were observed in prostate‐specific antigen (PSA) serum levels, IPSS (International Prostate Symptom Score), IIEF‐5 (International Index of Erectile Function), peak urinary flow rate (Qmax), postvoid residual volume (PVR), pad usage, incontinence, use of other drug classes, or surgeon's caseload.

Table 2 summarizes the results of the LRMs addressing persistent LUTS medication use after 24 months of follow‐up. In univariable models, the preoperative differences translated into significant associations for QoL (OR 1.27, *p* = 0.001), ICIQ‐SF (OR 1.05, *p* = 0.016), adiposity (OR 1.83, *p* = 0.018), indwelling catheter (OR 0.53, *p* = 0.014), ASA risk score III/IV (OR 2.05, *p* < 0.001), diabetes mellitus (OR 2.29, *p* = 0.003), and use of PDE‐5‐inhibitors (OR 3.06, *p* = 0.001). In the multivariable model, ASA score III/IV yielded an adjusted OR of 2.08 (*p* = 0.016), and preoperative use of PDE‐5‐inhibitors yielded an adjusted OR of 3.26 (*p* = 0.002), both independently associated with persistent LUTS medication use. QoL, ICIQ‐SF, adiposity, indwelling catheter, and diabetes mellitus failed to achieve independent predictor status. In a sensitivity analysis excluding PDE‐5‐inhibitor use from the multivariable LRM, ASA risk score III/IV remained the only independent predictor of persistent LUTS medication use (Table 3).

**TABLE 2:** Logistic regression models predicting persistent LUTS medication use after 24 months long follow‐up of 864 patients undergoing laser enucleation of the prostate at the University Hospital Frankfurt in the period from 11/2017 to 05/2023.

Preoperative characteristics	Univariable LRM			Multivariable LRM		
	OR	95% CI	*p*	adjusted OR	95% CI	*p*
Age (per year)	0.99	0.97–1.02	0.633	—	—	—
Prostate volume (per ccm)	1.00	1.00–1.00	0.945	—	—	—
PSA (per ng/ml)	0.98	0.94–1.02	0.268	—	—	—
IPSS storage (per point)	1.07	1.00–1.15	0.055	—	—	—
IPSS voiding (per point)	1.04	0.98–1.09	0.164	—	—	—
QoL (per point)	1.27	1.10–1.47	**0.001**	1.13	0.94–1.38	0.179
ICIQ‐SF (per point)	1.05	1.01–1.09	**0.016**	1.03	0.97–1.08	0.281
IIEF‐5 (per point)	0.99	0.96–1.02	0.610	—	—	—
Peak urinary flow rate (per ml/s)	1.00	0.99–1.01	0.794	—	—	—
Postvoid residual volume (per ml)	1.00	1.00–1.00	0.357	—	—	—
Adiposity (yes vs. no)	1.83	1.11–3.01	**0.018**	1.22	0.59–2.39	0.545
Indwelling catheter (yes vs. no)	0.53	0.32–0.88	**0.014**	0.65	0.28–1.35	0.251
Use of at least one pad per day (yes vs. no)	1.30	0.52–3.24	0.570	—	—	—
Incontinence^a^ (yes vs. no)	1.28	0.75–2.21	0.367	—	—	—
ASA risk score III/IV (vs. I/II)	2.05	1.36–3.09	**< 0.001**	2.08	1.13–3.78	**0.016**
Diabetes mellitus (yes vs. no)	2.29	1.32–3.99	**0.003**	1.06	0.46–2.26	0.931
Use of LUTS medication (yes vs. no)	1.92	0.90–4.10	0.135	—	—	—
Alphablockers	1.58	0.83–3.00	0.163	—	—	—
5‐AR‐inhibitors	1.22	0.66–2.27	0.530	—	—	—
Anticholinergics	2.04	0.85–4.91	0.111	—	—	—
Beta‐3‐agonists	4.49	0.40–104.79	0.187	—	—	—
PDE‐5‐inhibitors	3.06	1.56–5.98	**0.001**	3.26	1.48–6.91	**0.002**
Surgeon's caseload (per case)	1.00	1.00–1.00	0.334	—	—	—
Caseload over 200 (yes vs. no)	1.36	0.90–2.06	0.139	—	—	—

*Note:* Statistically significant values (*p* < 0.05) are shown in bold.

Abbreviations: AR = alphareductase; ASA = American Society of Anesthesiologists; CI = confidence interval; ICIQ‐SF = International Consultation on Incontinence Questionnaire Short Form; IIEF‐5 = International Index of Erectile Function; IPSS = International Prostate Symptom Score; LRM = logistic regression model; LUTS = lower urinary tract symptoms; OR = odds ratio; PDE = phosphodiesterase; PSA = prostate‐specific antigen; QoL = quality of life.

^a^ Continence defined as ICIQ‐SF ≤ 4 and no more than one security pad per day.

**TABLE 3:** Multivariable logistic regression models predicting persistent LUTS medication use after 24 months long follow‐up of 864 patients undergoing laser enucleation of the prostate at the University Hospital Frankfurt in the period from 11/2017 to 05/2023, without accounting for use of PDE‐5‐inhibitors.

Preoperative characteristics	Adjusted OR	95% CI	*p*
QoL (per point)	1.08	0.88–1.35	0.493
ICIQ‐SF (per point)	1.06	0.99–1.13	0.068
Adiposity (yes vs. no)	0.70	0.28–1.59	0.419
Indwelling catheter (yes vs. no)	0.67	0.26–1.51	0.365
ASA risk score III/IV (vs. I/II)	3.06	1.57–5.92	**< 0.001**
Diabetes mellitus (yes vs. no)	1.36	0.55–3.07	0.477

*Note:* Statistically significant values (*p* < 0.05) are shown in bold.

Abbreviations: ASA = American Society of Anesthesiologists; CI = confidence interval; ICIQ‐SF = International Consultation on Incontinence Questionnaire Short Form; LUTS = lower urinary tract symptoms; OR = odds ratio; PDE = phosphodiesterase; QoL = quality of life.

## 4 Discussion

Despite the growing body of evidence on the perioperative safety and functional efficacy of LEP, data on postoperative patterns of LUTS medication use and their preoperative predictors remain limited. To fill this void, we relied on our institutional tertiary‐care database of LEP patients and made several noteworthy observations.

First, we identified 864 patients with information on medication use within 24 months after LEP. This cohort size is smaller than that reported by Ory et al. and Sabharwal et al., who analyzed medication prescription in 1840 and 2549 LEP patients, respectively [6, 7]. However, their methodology relied on time‐interval assessments and prescription‐free survival analyses, which tends to overinterpret short‐term postoperative use of LUTS medication, as it cannot distinguish transient from persistent use. As a result, their methods do not allow valid conclusions about medication behavior at later follow‐up timepoints. In contrast, our dataset is the first to provide patient‐reported medication status over time, enabling a more accurate characterization of temporal use patterns and a more valid estimation of persistent LUTS medication use.

Second, the baseline characteristics of patients undergoing LEP at our institution aligned well with typical LEP populations. Median age was 70 years, median prostate volume was 80 ccm, and median PSA serum level was 4 ng/mL. In the current cohort, 30.9% of patients had a preoperative indwelling catheter and IPSS was 19. Moreover, baseline functional parameters indicated marked obstruction, with a median Qmax of 10 mL/s and a median PVR of 100 mL. All these values are consistent with existing literature, including a systematic review by Pallauf et al. reporting following mean preoperative values across 31 studies: age 69 years, prostate volume 61 mL, PSA serum level 3.2 ng/mL, indwelling catheter 30.1%, IPSS 23, Qmax 6 mL/s, mean PVR 133 mL [20, 21, 22, 23]. This alignment with previously reported LEP cohorts supports the external validity of the following findings.

Third, while 84.9% of patients reported preoperative use of LUTS medication, this proportion decreased to 12.6% of persistent use after 24 months of follow‐up. Alphablockers (80.9%) and 5‐AR‐inhibitors (13.8%) represented the predominant drug classes at baseline, whereas anticholinergics (4.6%), beta‐3‐agonists (0.3%), and PDE‐5‐inhibitors (1.8%) were infrequently used. These distributions align broadly with those reported by Sabharwal et al., who observed comparable preoperative rates for alphablockers (79.7%) and beta‐3‐agonists (0.9%), although their North American cohort demonstrated higher use of 5‐AR‐inhibitors (44.4%) and anticholinergics (18.4%) prior to LEP [6]. At 24 months, the rates of LUTS medication use in the current study had decreased markedly, reaching only minimal levels for most drug classes (alphablockers 1.8%, 5‐AR‐inhibitors 0%, anticholinergics 2.7%, and beta‐3‐agonists 0.4%), while PDE‐5‐inhibitors showed a modest increase relative to baseline (5.4%). The pronounced reduction in pharmacological treatment reflects the well‐known sustained functional benefit of LEP in relieving LUTS over the long term, thereby reducing the need for adjunctive medical therapy [7, 22]. In contrast, the increasing use of PDE‐5‐inhibitors may partly reflect indications outside the context of voiding physiology. Although our analysis was restricted to continuous use of long‐acting agents and excluded on‐demand regimens typically prescribed for erectile dysfunction, this indication remains relevant, as erectile dysfunction becomes more prevalent with age and may gain clinical importance once urinary symptoms have improved after LEP [17, 18].

Fourth, a transient increase in use of anticholinergics or beta‐3‐agonists was observed during the early postoperative period, peaking with 12% at 3 months after the procedure. This pattern is consistent with the well‐known occurrence of temporary irritative symptoms, which are common after LEP but tend to resolve spontaneously [9, 10, 11]. Our findings can be compared only to three previous studies. Kim et al. reported postoperative use of antispasmodic medication in 19% of patients, whereas Ory et al. and Sabharwal et al. documented rates of 14% and 12%, respectively [6, 7, 9]. In line with our observations, both Kim et al. and Sabharwal et al. reported that beta‐3‐agonists constituted only a fraction of this antispasmodic medication [6, 9]. These observations further support that short‐term postoperative antispasmodic therapy represents a transient phenomenon and should be interpreted separately from persistent long‐term LUTS medication use.

Fifth, in the current cohort, 12.6% of patients exhibited persistent LUTS medication use after 24 months of follow‐up after LEP, and several preoperative characteristics differentiated these patients from those who discontinued pharmacological therapy. Individuals with persistent LUTS medication use demonstrated worse baseline symptom scores and a higher prevalence of comorbidities, while they were less likely to present with an indwelling catheter. These descriptive differences were reflected in the univariable regression models, which showed significant associations for QoL (OR 1.27), ICIQ‐SF (OR 1.05), adiposity (OR 1.83), indwelling catheter (OR 0.53), ASA risk score III/IV (OR 2.05), diabetes mellitus (OR 2.29), and use of PDE‐5‐inhibitors (OR 3.06). The protective association of a preoperative indwelling catheter is consistent with previous observations suggesting that early decompression of a decompensated bladder may preserve detrusor function and facilitate earlier postoperative symptom improvement, thereby reducing the likelihood of requiring continued pharmacological therapy [24]. Conversely, patients presenting with greater preoperative symptom burden (high QoL and ICIQ‐SF scores) experience more residual symptoms postoperatively and therefore may have a higher likelihood of requiring continued LUTS medication [25]. Similarly, the associations with diabetes mellitus and adiposity align with previous reports linking both conditions to more pronounced LUTS and a higher prevalence of male urinary incontinence, which may contribute to persistent postoperative medication needs [26, 27].

Sixth, in the multivariable model, only comorbidity burden, reflected by an ASA score III/IV, and preoperative use of PDE‐5‐inhibitors remained independently associated with persistent LUTS medication use. This finding aligns with the observation that the distribution of drug classes used at 24 months after LEP is dominated by PDE‐5‐inhibitors (5.4%), which may also be prescribed for erectile dysfunction. The independent predictive value of preoperative use of PDE‐5‐inhibitor therefore appears consistent with the known relevance of erectile dysfunction in the aging male population [17, 18]. Moreover, symptomatic improvement following LEP may further heighten the clinical importance of erectile function, as enhanced quality of life could support a return to sexual activity. In addition, ASA score III/IV emerged as a further independent predictor, which is in accordance with previous reports linking higher comorbidity burden to impaired lower urinary tract function, reduced regenerative capacity, polypharmacy, and an increased likelihood of erectile dysfunction [26, 27, 28]. All of these may contribute to sustained postoperative medication use. A sensitivity analysis excluding PDE‐5‐inhibitor use confirmed that comorbidity burden remained the primary determinant of persistent postoperative LUTS medication use. This finding supports the robustness of the main results and further emphasizes the role of comorbidities in LUTS medication dependence after LEP.

Taken together, this first detailed longitudinal assessment of LUTS medication use after LEP demonstrates a substantial and sustained decline in medication reliance over 24 months. Only a small subset of patients continued LUTS medication, predominantly PDE‐5‐inhibitors. Preoperative use of PDE‐5‐inhibitors and a higher comorbidity burden, reflected by an ASA risk score III/IV, emerged as independent predictors of persistent LUTS medication use. The primary clinical implication of these findings is the improvement of preoperative patient counseling through more precise expectation management. Although for many patients a central motivation to undergo LEP is the expectation of becoming independent from LUTS medication, postoperative medication independence is likely but not guaranteed. This is particularly relevant in patients with a higher comorbidity burden or preoperative PDE‐5‐inhibitor use. In addition, some patients may require temporary initiation of antispasmodic medication in the early postoperative period before overall medication reliance declines. Importantly, the predictors of persistent LUTS medication use identified in the current study should not influence surgical indications but rather support individualized counseling regarding realistic expectations.

## 5 Limitations

Despite its novelty, this study is not without limitations. First, its retrospective design is inherently subject to selection and information bias. Although medication use was systematically recorded at predefined follow‐up timepoints, the data relied on patient‐reported intake rather than prescription records or pharmacy dispensing information. However, this approach also represents a methodological strength, as it avoids misclassification of medications that were prescribed but not actually taken by the patient [13]. Second, the study did not capture detailed information on postoperative indications for persistent pharmacological therapy. Consequently, it was not possible to differentiate whether ongoing medication reliance was directly related to persistent LUTS, prescribed for other urological indications, or initiated for non‐urological comorbidities during follow‐up. This limits the ability to determine whether persistent LUTS medication use reflects inadequate surgical response and cautions against interpreting it as definitive evidence of surgical failure. Given that this issue is particularly pertinent to PDE‐5‐inhibitor therapy, a sensitivity analysis excluding PDE‐5‐inhibitor use was conducted, which confirmed comorbidity burden as an independent risk factor. Third, follow‐up assessments were incomplete for a subset of patients, which may reduce the robustness of certain estimates despite the application of LOCF methods [14]. LOCF assumes stability of medication status over time and may therefore introduce bias. Accordingly, the results should be interpreted with appropriate caution. Fourth, the study was conducted at a tertiary‐care referral center, introducing the possibility of selection bias toward patients with higher comorbidity burden. Nevertheless, baseline characteristics remained consistent with those reported for typical LEP populations [20, 21, 22, 23].

## Author Contributions

M.F.: conceptualization, data curation, formal analysis, investigation, methodology, manuscript writing. M.I.T.: manuscript reviewing/editing. C.H.: manuscript reviewing/editing. M.J.M.: manuscript reviewing/editing. P.C.M.: manuscript reviewing/editing. L.A.K.: manuscript reviewing/editing. A.B.: manuscript reviewing/editing. F.K.‐H.C.: manuscript reviewing/editing. M.K.: conceptualization, project administration, supervision, manuscript reviewing/editing. All authors contributed to the study conception and design. The first draft of the manuscript was written by M.F. and all authors commented on previous versions of the manuscript. All authors read and approved the final manuscript.

## Funding

No funding was received for conducting this study. M.F. was supported by a personal fellowship from the Giersch Foundation.

## Disclosure

No material from other sources was reproduced in this manuscript. The authors did not use generative AI or AI‐assisted technologies in the development of this manuscript.

## Ethics Statement

Ethics approval was obtained from the Ethics Committee of the Faculty of Medicine at the University Hospital of the Goethe University Frankfurt (*Ethikkommission des Fachbereichs Medizin, Universitätsklinikum der Goethe‐Universität*; approval number E 98/21, 2021‐171). The study was conducted in accordance with the Declaration of Helsinki. Written informed consent to participate was obtained from all individual participants included in the study.

## Conflicts of Interest

A.B., F.K.‐H.C., and M.K. are proctors for Olympus and Boston Scientific.

## Data Availability

The data that support the findings of this study are available from the corresponding author upon reasonable request.
